# Immunoregulatory Therapy Improves Reproductive Outcomes in Elevated Th1/Th2 Women with Embryo Transfer Failure

**DOI:** 10.1155/2022/4990184

**Published:** 2022-06-26

**Authors:** Shihui Meng, Tianzhen Zhang, Chun Li, Xiaowei Zhang, Huan Shen

**Affiliations:** ^1^Reproductive Medical Center, Department of Obstetrics and Gynecology, Peking University People's Hospital, Peking University, Beijing, China; ^2^Department of Rheumatology & Immunology, Clinical Immunology Center of Peking University People's Hospital, Beijing, China; ^3^Department of Urology, Peking University People's Hospital, Peking University, Beijing, China

## Abstract

**Objective:**

Immunological disturbance is one of the crucial factors of implantation failure. Limited data exists evaluating immunoregulatory therapy in patients with implantation failures.

**Methods:**

This is a retrospective cohort study on patients who had failed embryo transfer cycle and had elevated Th1/Th2 cytokine ratios between 1/2019 and 3/2020. Patients were assigned into two groups based on whether they received immunoregulatory treatment during a frozen transfer cycle. The primary outcome was live birth rate. Secondary outcomes included clinical pregnancy, implantation rate, and neonatal outcomes.

**Results:**

Of 71 patients enrolled, 41 patients received immunoregulatory therapy and 30 patients did not. Compared to untreated patients, rate of live birth was significantly elevated in the treated group (41.5% vs. 16.7%, *P* = 0.026). Rate of biochemical pregnancy, implantation, clinical pregnancy, and ongoing pregnancy between two groups were 56.1% vs. 40% (*P* = 0.18), 36.5% vs. 23.9% (*P* = 0.15), 51.2% vs. 30% (*P* = 0.074), and 41.5% vs. 16.7% (*P* = 0.03), respectively. Although there was no statistical significance, women receiving treatment also had a tendency of lower frequency of pregnancy loss (19.0% vs. 44.4%, *P* = 0.20). No adverse events were found between newborns of the two groups. Immunoregulatory therapy, age, infertility type, ovulation induction protocol, number of oocytes retrieved, artificial cycle embryo transfer, and cleavage transfer were associated with live birth in univariate analysis (all *P* < 0.05). Only immunoregulatory therapy was associated with live birth after adjustment of confounders (OR = 5.02, 95% CI: 1.02-24.8, *P* = 0.048).

**Conclusions:**

Immunoregulatory therapy improves reproductive outcomes in elevated Th1/Th2 cytokine ratio women with embryo transfer failure.

## 1. Introduction

Incidence of implantation failures varies from 8 to 33% in the general population [1]. When conducting IVF/ET, pregnancy is established when an embryo, which is a semiallograft, is successfully implanted to the maternal decidua with an establishment of maternal immune tolerance [2]. The disturbance of Th1 and Th2 cytokines may result in implantation failure. Elevated levels of Th1 cells are associated with embryo rejections, whereas elevated Th2 cell levels are associated with successful pregnancy [3]. Cytokines produced by Th1 cells, such as TNF-*α*, promote inflammatory and thrombotic responses. Cytokines produced by Th2 cells such as IL-4 inhibit Th1 cell-induced tissue factors. Previous studies have also shown significantly higher Th1/Th2 ratios in peripheral blood samples in patients with implantation failures [4].

Immunoregulatory therapy may be effective in treating immune disturbance. Prednisone (PDN), hydroxychloroquine (HCQ), or cyclosporine (CsA) has been proven to inhibit Th1 cytokine secretion, increase the number of regulatory T cells, and induce maternofetal tolerance [5]. Therefore, use of immunoregulators prior to embryo transfer may improve the IVF outcome. However, some studies showed adverse results [6]. These studies did not target on well-selected patients with immune disturbance which may present as an elevated Th1/Th2 cell ratio. Besides, these studies did not use combination of immunoregulatory medicines. Therefore, we conducted a retrospective cohort study to investigate the reproductive outcomes of FET (frozen-thawed embryo transfer) cycle after use of immunoregulatory therapy versus no treatment in women with previous implantation failure and an elevated peripheral blood Th1/Th2 cell ratio. A preprint has previously been published [7].

## 2. Methods

### 2.1. Study Population

This is a retrospective cohort study in which patients were enrolled in Reproductive Medicine Department, Peking University People's Hospital between January 2019 and March 2020. We enrolled patients based on the following eligibility criteria: (1) patients age < 40 years, (2) having at least one failed embryo transfer cycle previously, (3) having an elevated peripheral blood Th1/Th2 ratio, and (4) planning to undergo frozen-thawed embryo transfer. Patients were excluded if they had any structural lesions of the uterus or hydrosalpinges. Demographic and baseline clinical data were abstracted from the clinical records. All serum laboratory values were obtained in our laboratory system. Patients who received immunoregulatory therapy were enrolled into the treated group, and those who did not receive immunoregulatory therapy were enrolled into the nontreated group.

The study was approved by the institutional ethics review committee (2018PHB141-01). A signed informed consent form was obtained from all patients prior to treatment.

### 2.2. Analyses of the Peripheral Blood Th1/Th2 Cells

According to Kwak-Kim et al. [4], mean TNF-*α*/IL-4 level is 12.81 ± 2.52 in patients with previous implantation failures; we took one standard deviation plus mean value, which is 15.33, as a lower limit of the elevated Th1/Th2 cytokine ratio. Therefore, the elevated Th1/Th2 ratio was defined as TNF-*α*/IL-4 equal to 15.33 or above. To evaluate the value of Th1/Th2 ratios, peripheral blood was drawn between cycle days (CD) 3 and 9 of this ART cycle. TNF-*α* and IL-4 concentrations in serum were measured using ELISA kits from BioLegend (San Diego, CA, USA), according to the manufacturer's instruction. Serum was prepared by centrifugation of coagulated blood tubes at 2000g for 10 min at room temperature and stored in −70°C. Samples were tested for IL-4 and TNF-*α* using a sandwich enzyme-linked immunosorbent assay according to the manufacturer's instructions (R&D Systems, USA). Their concentrations were calculated using standard curves.

### 2.3. Immunosuppressive Treatment

Patients with only one time of implantation failure received prednisone only. Patients who had two or more implantation failures took combination of prednisone, hydroxychloroquine, and cyclosporine. Patients who had concerns about safety of cyclosporine received combination of prednisone and hydroxychloroquine. Cyclosporine is a strong immunosuppressant which can cause serum creatinine and urea nitrogen elevation, as well as discomforts after the first contact. An animal test has shown no teratogenic risk but needs further clinical verifications. We explained its benefits and risks to all patients before treatment.

5 mg prednisone daily was begun on the first menstrual day of the FET cycle and continued until the 8 weeks of gestation. 200 mg hydroxychloroquine daily was started from day one of the FET cycle and continued until 8 weeks of gestation. 100 mg daily cyclosporine was begun on the day of embryo transfer and continued until the 8 weeks of gestation.

### 2.4. FET Procedures

All patients received standardized ovarian stimulation regimens, oocyte retrieval, and fertilization, followed by a planned frozen transfer of up to two day-3 or day-5 embryos. Patients received one of following regimens based on individual situations: gonadotropin-releasing hormone (GnRH) antagonist, GnRH agonist long protocol, and mild stimulation protocol. When at least two follicles reached 18 mm, 5,000 to 10,000 IU of hCG (Covidrel, Merck Serono) was administered and oocyte retrieval occurred 36 hours later. Luteal phase support was started from the day of ovulation with oral dydrogesterone at a dose of 20 mg twice a day and was continued until the day of serum hCG testing. Up to two cleavage stage frozen embryos or day 5 trophoblasts were thawed and transferred, respectively. The pregnancy test will be performed 2 weeks after embryo transfer. In women with a positive hCG test, luteal phase support was continued until 10 weeks of gestation.

### 2.5. Outcome Measurements

The primary outcome was live birth rate, which was defined as the delivery of any viable neonate who was 28 weeks of gestation or older. Secondary outcomes included biochemical pregnancy, clinical pregnancy, implantation rate, and neonatal outcomes.

### 2.6. Statistical Analysis

Baseline characteristics and laboratory results were summarized for two groups utilizing descriptive statistics, including percentage, means ± standard deviation (SD), and 95% CI. For the quantitative variable, the *t*-test was used to compare group differences. For categorical variables, the chi-square test or Mann–Whitney *U* test was used for group comparisons. Significance level was set at *P* < 0.05; all data were analyzed by using SPSS 23.0 (SPSS, IBM, NYU).

## 3. Results

### 3.1. Study Population

**Figure 1:**
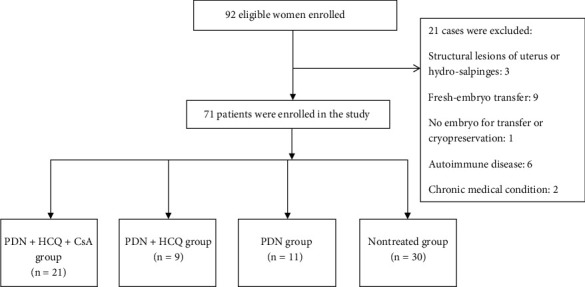
Disposition of patients.

Ninety-two patients were screened, and 21 were excluded for structural lesions of the uterus or hydrosalpinges, fresh embryo transfer, no embryo for transfer or cryopreservation, autoimmune disease, and chronic medical condition. Seventy-one patients met the eligibility and were enrolled into the study (Figure 1). Forty-one patients who received immune therapy to regulate immune disturbance were enrolled into the treatment group, and 30 patients who did not receive immunotherapy were enrolled into the control group. Of 41 patients in the treatment group, 30 patients who had twice or more implantation failure received combination therapy (prednisone, hydroxychloroquine with/without cyclosporine), and 11 patients who had one time of implantation failure received prednisone.

The mean age of the treated group was 34.61 ± 4.05 years, comparable to 4.57 ± 3.42 years of the nontreated group. The mean duration of infertility was 3.88 ± 2.27 years in the treated group and 4.37 ± 2.99 years in the nontreated group. Unexplained infertility was the most indications of all patients, accounting for 36.6% in the treated group and 36.7% in the nontreated group. On average, the treated group had 2.34 ± 1.44 failed embryo transfer cycles and 4.44 ± 2.79 failed transferred embryos; the nontreated group had 1.97 ± 1.35 failed embryo transfer cycles and 3.73 ± 2.84 failed transferred embryos. In this IVF cycle, 34.1% of treated patients received GnRH agonists, 41.5% received GnRH antagonists, and 24.4% received mild stimulation. 33.3% of nontreated patients received GnRH agonists, 43.3% received GnRH antagonists, and 23.3% received mild stimulation. No differences were found between the treated group and nontreated group in age, BMI, fertility histories, and indications for IVF (Table 1). There were also no statistical significances of outcomes of controlled ovarian hyperstimulation. The mean Th1/Th2 ratio was 29.42 ± 12.90 in the treated group and 29.71 ± 14.87 in the control group, which had no statistical significances (*P* = 0.086). However, treated patients had significantly higher Th17 levels than nontreated patients (1.92 ± 0.85 vs. 1.35 ± 0.36, *P* = 0.001, *t* = 0.57).

### 3.2. Live Birth and Neonatal Outcomes

The mean number of embryos transferred was higher in the treated group than in the control group (1.8 ± 0.5 vs. 1.5 ± 0.5, *P* = 0.02). Other variables in frozen embryo transfer procedures did not have statistical significances (Table 2). The rate of live birth was 41.5% in all treated patients (17 patients), higher than 16.7% of nontreated patients (5 patients, *P* = 0.026). The treated group had a tendency of higher frequency of implantation, clinical pregnancy, and ongoing pregnancy (56.1% vs. 40%, *P* = 0.18; 36.5% vs. 23.9%, *P* = 0.15; 51.2% vs. 30%, *P* = 0.074; and 41.5% vs. 16.7%, *P* = 0.03). Among the treated group, patients treated with combination of prednisone, hydroxychloroquine, and cyclosporine had the highest live birth rate of 52.4% (11 patients), patients treated with prednisone and hydroxychloroquine had the second highest live birth rate (33.3%, patients), and patients treated with only prednisone had the lowest live birth rate of 27.3% (3 patients). Women receiving treatment also had a tendency of lower frequency of pregnancy loss (19.0% vs. 44.4%, *P* = 0.20), indicating the establishment of immune tolerance in the embryo-maternal interface. No congenital abnormalities or other complications were found on newborns between the two groups, suggesting safety of immunoregulatory therapy. Detailed information about newborns is shown in Supplementary Table 1.

### 3.3. Risk Factors Associated with Live Birth in Univariate Analysis

As determined by univariate analysis (Table 3), age (OR = 0.78, 95% CI: 0.66-0.92, *P* = 0.02), secondary infertility (OR = 0.18, 95% CI: 0.06-0.57, *P* = 0.02), and cleavage transfer (OR = 0.21, 95% CI: 0.06-0.71, *P* = 0.01) were negatively related to live birth. Number of oocytes retrieved (OR = 1.13, 95% CI: 1.04-1.23, *P* < 0.01), artificial cycle embryo transfer (OR = 2.22, 95% CI: 0.56-8.82, *P* < 0.01), GnRH agonist protocol (OR = 16, 95% CI: 1.82-140.55, *P* = 0.01), and immunoregulatory therapy (OR = 3.91, 95% CI: 1.25-12.25, *P* = 0.02) were positively associated with live birth. In the stratification of immunoregulatory therapy, PDN+HCQ+CsA was significantly associated with live birth (OR = 6.67, 95% CI: 1.83-24.26, *P* < 0.01), while PDN+HCQ (*P* = 0.95) and prednisone (*P* = 0.7) had no statistical significance.

### 3.4. Relationship between Immunoregulatory Therapy and Live Birth in Multivariable Logistic Regression Models

**Figure 2:**
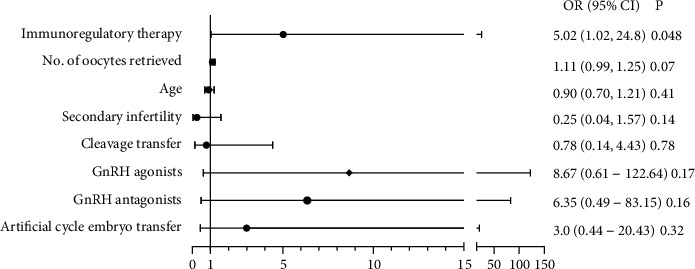
Logistic regression model evaluating the relationship between live birth and immunoregulatory therapy.

To further investigate the relationship between immunoregulatory therapy and live birth, we conducted multivariate analysis (Table 3). We adjusted significant risk factors in univariate analysis including age, secondary infertility, cleavage transfer, number of oocytes retrieved, artificial cycle embryo transfer, and GnRH agonist protocol. Immunoregulatory therapy was positively associated with live birth (OR = 5.02, 95% CI: 1.02-24.8, *P* = 0.048) (Figure 2).

## 4. Discussion

In this study, we reported on data of immunotherapy therapy for improving IVF-ET outcomes in patients with the elevated peripheral Th1/Th2 cytokine ratio. To our knowledge, this is the first study to evaluate the efficacy and safety of a combination of immunoregulators to IVF-ET in this special population. Our results indicated that use of prednisone, hydroxychloroquine, or prednisone during the frozen embryo transfer cycle for patients with the elevated peripheral cytokine Th1/Th2 ratio improved live birth rate compared to those untreated. It is worth noting that live birth rate of all patients who underwent frozen embryo transfer is 32.5% in out center; use of an immunoregulator raised live birth rate to the above average level. Therefore, immunoregulatory therapy can bring benefits to the clinical practice in treating implantation failures.

Studies found that implantation failure is associated with elevated Th1/Th2 [8]. Cytokines of Th1 and Th2 cells interfere with pregnancy through several mechanisms. Cytokines of Th1 cells such as TNF-*α* may activate macrophages which could attack the trophoblast and trigger processes at the maternal utero-placental blood vessels by activation of vascular endothelial cell procoagulant [9]. In contrast, Th2 cytokines such as IL-4 inhibit Th1-induced tissue factor production by monocytes. In our study, the mean Th1/Th2 (TNF-*α*/IL-4) ratio was 29.42 ± 12.90 in the treated group, which was much higher than the Th1/Th2 ratio of both infertile patients (2.4 ± 0.4, *n* = 80) [4] and patients with IVF failures (12.81 ± 2.52, *n* = 9) in other studies [10], suggesting a severer infertile condition in our population. Rate of live birth improved since the increase in the type of immunoregulators, and combination of prednisone, hydroxychloroquine, and cyclosporine had the most favorable outcome. But this trend needs to be verified in the larger population. Prednisone, hydroxychloroquine, and cyclosporine have different mechanisms in regulating immune status; the crosstalk among them may have enhanced effect on the cytokine network and result in better outcomes. This also suggested that elevated Th1/Th2 ratios should be used to identify targeted patients who were suitable for immunoregulatory therapy.

Limited data exists evaluating live birth rate of IVF-ET patients with immune disturbance after immunoregulatory therapy. In previous studies, prednisone was usually combined with hydroxychloroquine and other immunoregulatory regimens to improve the natural conception rate of women with disease of the immune system such as obstetrical antiphospholipid syndrome and systemic lupus erythematosus [11]. Those medications were also proven effective in immune disturbances. A study showed that hydroxychloroquine administration in women with RIF with a high TNF-*α*/IL-10 ratio significantly decreased serum level of TNF-*α* and significantly increased serum level of IL-10 (*P* < 0.0001) [5]. Cyclosporine A (CsA) was also proven to increase Th2-associated responses (*P* = 0.0001) and reduce Th1/Th2 (26.71 ± 7.32 vs. 18.56 ± 4.92, *P* < 0.0001) in women with recurrent pregnancy loss [12]. However, reproductive outcomes were not analyzed in either study. Dan and Hong [13] analyzed reproductive outcomes after prednisolone administration during assisted reproductive technology (ART). The result had no significance (pregnancy rate: RR 1.02, 95% CI 0.84-1.24; clinical pregnancy rate: RR 1.01, 95% CI 0.82-1.24; and implantation rate: RR 1.04, 95% CI 0.85-1.28). Reasons may be because these studies did not subanalyze patients with immune abnormality. Therefore, immunoregulatory therapy may be effective in patients with immune disturbance, and the Th1/Th2 cytokine ratio should be used to identify targeted patients. This study provides a possible way of treating implantation failure in clinical practice. Certain patients could try to receive immunoregulatory therapy to improve reproductive outcomes and reduce time of embryo transfer.

Safety of immunoregulatory therapy needs to be emphasized. Prednisolone has long been proven safe in pregnancy with low prednisone amounts (<10 mg/day) [14]. The safety of hydroxychloroquine is also well established with a favorable safety profile [15]. CsA is a particularly lipophilic peptide being able to inactively traverse the placenta and enter the fetal circulation. Though drug has been found in the placenta, cord blood, and amniotic fluid [16], it has not been convincingly verified whether or not it interferes with fetus development and growth. A meta-analysis implied that CsA does not seem to be a major human teratogen [17]. Other researches have also displayed that the use of CsA during pregnancy does not increase the threat for inherited defects in infants [18].

This study has limited statistical power due to small sample size. A prospective randomized study is needed in the future to examine the efficacy and safety of immunotherapy. Besides, treated patients had higher Th17 levels which induce inflammation, suggesting a severer situation of immune abnormality compared with the control group [19]. However, Treg cells express anti-inflammatory cytokines; recent studies have indicated that the balance between Treg and Th17 cells is important for maintaining a normal pregnancy. Therefore, the Th17/Treg ratio should also be analyzed. After two times of implantation failure, we used a combination of immunotherapy. The efficacy of single medication should be analyzed in the further study.

In conclusion, immunoregulatory therapy improves reproductive outcomes in elevated Th1/Th2 cytokine ratio women with embryo transfer failure.

## Figures and Tables

**Table 1:** Baseline characteristics of patients with elevated Th1/Th2 ratios who were treated with immunoregulators and without immunoregulators.

Variable	PDN+HCQ+CsA treated (*n* = 21)	PDN+HCQ treated (*n* = 9)	PDN treated (*n* = 11)	All treated (*n* = 41)	Nontreated (*n* = 30)	*P* value (all treated vs. nontreated)
Age (y)	33.67 ± 3.99	34.67 ± 4.03	36.36 ± 3.93	34.61 ± 4.05	34.57 ± 3.42	*P* = 0.96, *t* = 0.05
Body mass index (kg/m^2^)	22.59 ± 2.88	23.85 ± 2.76	20.95 ± 2.36	22.42 ± 2.85	23.83 ± 4.56	*P* = 0.12, *t* = 1.57
Fertility history						
Duration of infertility (y)	4.24 ± 2.19	3.05 ± 2.13	3.86 ± 2.55	3.88 ± 2.27	4.37 ± 2.99	*P* = 0.43, *t* = 0.79
Previous conception, no. (%)	7 (33.3)	6 (66.7)	5 (45.5)	18 (43.9)	19 (63.3)	*P* = 0.11, *χ*^2^ = 2.62
Spontaneous abortion, no. (%)	5 (23.8)	4 (44.4)	1 (9.1)	10 (24.4)	10 (33.3)	*P* = 0.41, *χ*^2^ = 0.69
Indications for IVF, no. (%)						
Unexplained infertility	8 (38.1)	2 (11.1)	5 (45.5)	15 (36.6)	11 (36.7)	*P* = 0.994, *χ*^2^ = 0.00
Tubal factor	3 (14.3)	6 (66.7)	2 (18.2)	11 (26.8)	9 (30.0)	*P* = 0.769, *χ*^2^ = 0.086
Male factor	3 (14.3)	2 (22.2)	1 (9.1)	6 (14.6)	4 (13.3)	*P* = 0.876, *χ*^2^ = 0.024
Ovulatory factor	5 (23.8)	2 (22.2)	2 (18.2)	9 (22.0)	5 (16.7)	*P* = 0.580, *χ*^2^ = 0.306
Previous embryo transfer history, no.						
Number of failed embryo transfer cycles	3.05 ± 1.43	1.44 ± 0.88	1.18 ± 0.60	2.34 ± 1.44	1.97 ± 1.35	*P* = 0.27, *t* = 1.11
Total number of transferred embryos	5.81 ± 2.79	3.89 ± 2.53	2.09 ± 1.14	4.44 ± 2.79	3.73 ± 2.84	*P* = 0.3, *t* = 1.04
Th1/Th2 ratio	31.09 ± 14.32	31.01 ± 12.75	24.92 ± 9.76	29.42 ± 12.90	29.71 ± 14.87	*P* = 0.086, *t* = 0.932
Th17	1.99 ± 0.90	1.6 ± 0.43	2.06 ± 0.99	1.92 ± 0.85	1.35 ± 0.36	*P* = 0.001, *t* = −0.57
Ovulation induction protocol, no. (%)						
GnRH agonists	7 (33.3)	3 (33.3)	4 (36.4)	14 (34.1)	10 (33.3)	*P* = 0.987, *Z* = 0.026
GnRH antagonists	9 (42.9)	3 (33.3)	5 (45.5)	17 (41.5)	13 (43.3)	
CC mild stimulation	5 (23.8)	3 (33.3)	2 (18.2)	10 (24.4)	7 (23.3)	
No. of oocytes retrieved	12.74 ± 6.98	13.38 ± 11.02	14.75 ± 9.32	13.38 ± 8.45	13.31 ± 8.08	*P* = 0.973, *t* = 0.035

Note: values are presented as mean ± SD or frequencies (percentage); Th1 cell: tumor necrosis factor alpha-producing T helper cell (CD3^+^/4^+^/TNF-*α*^+^); Th2 cell; IL-4-producing T helper cell (CD3^+^/4^+^/IL-4^+^).

**Table 2:** Frozen-thawed embryo transfer and reproductive outcomes of patients treated with immunoregulators and without immunoregulators.

Variable	PDN+HCQ+CsA treated (*n* = 21)	PDN+HCQ treated (*n* = 9)	PDN treated (*n* = 11)	All treated (*n* = 41)	Nontreated (*n* = 30)	*P* value (all treated vs. nontreated)
Regimen of endometrial preparation						
Natural cycle no. (%)	3 (14.3)	2 (22.2)	2 (18.2)	7 (17.1)	8 (26.7)	*P* = 0.33, *χ*^2^ = 0.96
Artificial cycle no. (%)	18 (85.7)	7 (77.8)	9 (81.8)	34 (82.9)	22 (73.3)	
Endometrium thickness before embryo transfer (mm)	9.0 ± 2.0	8.8 ± 2.0	9.4 ± 2.0	9.1 ± 1.9	8.8 ± 1.5	*P* = 0.57, *t* = 0.58
Type of embryo transferred						
Cleavage transfer, no. (%)	6 (28.6)	4 (44.4)	5 (45.5)	15 (36.6)	12 (40.0)	*P* = 0.77, *χ*^2^ = 0.09
Blastocyst transfer, no. (%)	15 (71.4)	5 (55.6)	6 (54.5)	26 (63.4)	18 (60.0)	
No. of embryos transferred						
Mean	1.8 ± 0.5	1.8 ± 0.4	1.9 ± 0.3	1.8 ± 0.5	1.5 ± 0.5	*P* = 0.02, *t* = 2.35
One embryo, no./total no. (%)	6 (28.6)	2 (22.2)	1 (9.09)	9 (22.0)	14 (46.7)	*P* = 0.77, *χ*^2^ = 0.09
Two embryos, no./total no. (%)	15 (71.4)	7 (77.8)	10 (90.9)	32 (78.0)	16 (53.3)	
No. of MGE transferred	1.4 ± 0.5	1.6 ± 0.7	1.4 ± 0.8	1.4 ± 9.1	1.2 ± 0.7	*P* = 0.90, *t* = 0.37
Reproductive outcomes						
Biochemical pregnancy, no. (%)	14 (66.7)	3 (33.3)	6 (54.5)	23 (56.1)	12 (40)	*P* = 0.18, *χ*^2^ = 1.80
Implantation rate, no./total no. (%)	17/37 (45.9)	5/16 (31.3)	7/21 (33.3)	27/74 (36.5)	11/46 (23.9)	*P* = 0.15, *χ*^2^ = 2.07
Clinical pregnancy, no. (%)	12 (57.1)	3 (33.3)	6 (54.5)	21 (51.2)	9 (30.0)	*P* = 0.07, *χ*^2^ = 0.20
Ongoing pregnancy, no. (%)	11 (52.4)	3 (33.3)	3 (27.3)	17 (41.5)	5 (16.7)	*P* = 0.03, *χ*^2^ = 4.98
Live birth, no. (%)	11 (52.4)	3 (33.3)	3 (27.3)	17 (41.5)	5 (16.7)	*P* = 0.03, *χ*^2^ = 4.98
Pregnancy loss among clinical pregnancies, no. (%)	1 (8.3)	0	3	4 (19%)	4 (44.4%)	*P* = 0.20, *χ*^2^ = 2.08

CC: clomiphene citrate; MGE: morphologically good-quality embryos.

**Table 3:** Logistic regression models evaluating the relationship between live birth and immunoregulatory therapy.

Variable	Univariate analysis		Multivariate analysis		
	OR	95% CI	OR	95% CI	*P*
Age (y)	0.78	0.66-0.92	0.9	0.7-1.21	0.41
Body mass index	0.96	0.83-1.11	—		
Duration of infertility	1.09	0.90-1.31	—		
Secondary infertility	0.18	0.06-0.57	0.25	0.04-1.57	0.14
Unexplained infertility	0.5	0.17-1.48	—		
Tubal factor	0.86	0.28-2.63	—		
Male factor	1.47	0.37-5.84	—		
Ovulatory factor	2.56	0.78-8.48	—		
Recurrent implantation failure	1.63	0.46-5.82	—		
Immunoregulatory therapy	3.91	1.25-12.25	5.02	1.02-24.8	0.048
PDN+HCQ+CsA	6.67	1.83-24.26	—		
PDN+HCQ	2.5	0.46-13.50			
PDN	1.88	0.36-9.65			
Follicle-stimulating hormone (IU/liter)	0.8	0.61-1.05	—		
Luteinizing hormone (IU/liter)	0.97	0.8-1.18	—		
Estradiol (pg/mL)	0.99	0.97-1.02	—		
Progesterone (nmol/L)	1.12	0.23-5.46	—		
Total testosterone (ng/mL)	1.04	0.5-2.19	—		
Th1/Th2 ratio	1.02	0.98-1.05	—		
Th17	1.18	0.61-2.29	—		
Ovulation induction protocol					
GnRH agonists, no. (%)	16	1.82-140.55	8.67	0.61-122.64	0.11
GnRH antagonists, no. (%)	8	0.92-69.24	6.35	0.49-83.15	0.16
CC mild stimulation, no. (%)	Reference	Reference	Reference	Reference	Reference
No. of oocytes retrieved	1.13	1.04-1.23	1.11	0.99-1.25	0.07
Artificial cycle embryo transfer	2.22	0.56-8.82	3.00	0.44-20.43	0.32
Endometrium thickness before embryo transfer (mm)	1.02	0.77-1.37	—		
Cleavage transfer, no. (%)	0.21	0.06-0.71	0.78	0.14-4.43	0.78
Blastocyst transfer, no. (%)	Reference	Reference	Reference	Reference	Reference
No. of embryos transferred	0.96	0.80-1.15	—		
No. of MGE transferred	1.39	0.65-2.99	—		

Note: values are presented as mean ± SD or frequencies (percentage). Th1 cell: tumor necrosis factor alpha-producing T helper cell (CD3^+^/4^+^/TNF-*α*^+^); Th2 cell; IL-4-producing T helper cell (CD3^+^/4^+^/IL-4^+^); CC: clomiphene citrate; MGE: morphologically good-quality embryos.

## Data Availability

The datasets used and/or analyzed during the current study are available from the corresponding author on reasonable request.

## Abbreviations

IVF-ET: In vitro fertilization and embryo transfer
PDN: Prednisone
HCQ: Hydroxychloroquine
CsA: Cyclosporine
CC: Clomiphene citrate
MGE: Morphologically good-quality embryos.

## Supplementary Materials

Supplementary Materials Supplementary Table 1: neonatal information of treated patients and nontreated patients (see supplementary files).
