# Supplementary material for: Immunoregulatory Therapy Improves Reproductive Outcomes in Elevated Th1/Th2 Women with Embryo Transfer Failure
Source: Biomed Res Int. 2022 Jun 26;2022:4990184. doi: 10.1155/2022/4990184 (PMC9251089; doi:10.1155/2022/4990184)
Supplement: Supplementary Materials — Supplementary Table 1: neonatal information of treated patients and nontreated patients (see supplementary files). [file 4990184.f1.docx]

Supplemental table 1. Neonatal outcomes of treated patients and non-treated patients

| Group | Patients | Number of newborns | Mode of delivery | Gestational days to delivery (weeks+days) | Birthweight (gm)a | Height  (cm)a | Sex of newborn | Complications |
| --- | --- | --- | --- | --- | --- | --- | --- | --- |
| All Treated | 1 | 1 | Cesarean section | 38 | 3830 | 52 | girl | no |
|  | 2 | 1 | Cesarean section | 39+4 | 3800 | 51 | boy | no |
|  | 3 | 1 | Cesarean section | 40 | 3220 | 48 | boy | no |
|  | 4 | 1 | Vaginal delivery | 38+6 | 3300 | 50 | boy | no |
|  | 5 | 1 | Cesarean section | 40+2 | 3400 | 52 | girl | no |
|  | 6 | 1 | Cesarean section | 39 | 3520 | 51 | girl | no |
|  | 7 | 1 | Cesarean section | 39+6 | 3650 | 53 | boy | no |
|  | 8 | 1 | Cesarean section | 39+5 | 3650 | 53 | boy | no |
|  | 9 | 1 | Vaginal delivery | 39+5 | 3760 | 50 | boy | no |
|  | 10 | 1 | Cesarean section | 33 | 2800 | 48 | girl | cervical incompetence |
|  | 11 | 1 | Cesarean section | 39+3 | 3000 | 50 | boy | no |
|  | 12 | 1 | Vaginal delivery | 38+6 | 3300 | 50 | girl | no |
|  | 13 | 2 | Cesarean section | 36+4 | 2250/2350 | 46/44 | boy/girl | no |
|  | 14 | 2 | Cesarean section | 36+2 | 2300/2100 | 45/44 | boy/girl | no |
|  | 15 | 2 | Cesarean section | 37+6 | 2700/2400 | 51/47 | boy/girl | no |
|  | 16 | 1 | Cesarean section | 38+1 | 3050 | 49 | boy | no |
|  | 17 | 1 | Vaginal delivery | 40+1 | 3600 | 49 | girl | no |
| Nontreated | 1 | 2 | Cesarean section | 36 | 2650/2740 | 44/43 | boy/girl | no |
|  | 2 | 2 | Cesarean section | 36 | 2645/2900 | 46/45 | girl/boy | no |
|  | 3 | 1 | Vaginal delivery | 40+5 | 3470 | 51 | girl | no |
|  | 4 | 1 | Cesarean section | 34+6 | 1890 | 45 | girl | no |
|  | 5 | 2 | Cesarean section | 37+3 | 3085/3215 | 50/49 | boy/girl | no |

aTTN, transient tachypnea of newborn
